# Effects of hatching system on the welfare of broiler chickens in early and later life

**DOI:** 10.1016/j.psj.2020.12.043

**Published:** 2020-12-23

**Authors:** Mona F. Giersberg, Roos Molenaar, Ingrid C. de Jong, Carol Souza da Silva, Henry van den Brand, Bas Kemp, T. Bas Rodenburg

**Affiliations:** ∗ Adaptation Physiology Group, Wageningen University & Research, 6700 AH Wageningen, the Netherlands; † Animals in Science and Society, Department of Population Health Sciences, Faculty of Veterinary Medicine, Utrecht University, 3508 TD Utrecht, the Netherlands; ‡ Wageningen Livestock Research, Wageningen University & Research, 6700 AH Wageningen, the Netherlands

**Keywords:** broiler, early feeding, on-farm hatching, welfare, behavior

## Abstract

Broiler chicks usually hatch in the hatchery without access to feed and water until placement at the farm. This can affect their health and welfare negatively. Therefore, alternative strategies have been developed, for instance providing chicks with early nutrition in the hatchery or hatching eggs directly on-farm. However, information on the physical and mental welfare of chicks hatched in these systems compared to conventionally hatched chicks is limited. The aim of this study was to investigate the effects of alternative hatching systems on the welfare of broiler chickens in early and later life. A system comparison was performed with chickens that hatched conventionally in a hatchery (**HH**), in a system which provided light, feed, and water in a hatcher (hatchery-fed, **HF**), or on-farm (on-farm hatched, **OH**, where feed and water were available and transport of day-old chicks from the hatchery to the farm was not necessary). Chickens were reared in 3 batches, in 12 floor pens per batch (approximately 1,155 animals per pen), with a total of 12 replicates per treatment. Animal-based welfare indicators were assessed following standard protocols: plumage cleanliness, footpad dermatitis (**FPD**), hock burn, skin lesions (all at day 21 and 35 of age), and gait score (day 35). Furthermore, a set of behavioral tests was carried out: novel environment (day 1 and 21), tonic immobility, novel object, and avoidance distance test (day 4 and 35). Plumage cleanliness, hock burn, and skin lesions were affected by age but not by hatching system, with older broilers scoring worse than younger ones (*P* < 0.05). An effect of hatching system was only found for FPD, with the highest prevalence in HH chickens, followed by HF and OH chickens (*P* < 0.05). All responses measured in the behavioral tests were affected by age but not by hatching system. In later life, chickens were significantly less fearful than during the first days of life. The results indicate that conventionally hatched chickens scored significantly worse for FPD, whereas, in general, hatching system seemed to have minor effects on other broiler welfare indicators.

## Introduction

In commercial hatcheries, broiler chicks usually hatch in conventional hatchers, where they are not provided with feed and water until placement at the farm. Chicks from 1 batch of eggs do not hatch at exactly the same time but do so within a hatch window, which is defined as the time interval from the first to the last chick hatched. Depending on incubation conditions and breeder flock characteristics (Lourens et al., 2005), the hatch window ranges between 24 and 48 h (Careghi et al., 2005). The fasting period of the chicks is further increased by the time required for processing and handling procedures at the hatchery (e.g., selection of second grade chicks and vaccination), the duration of transport, and unloading at the broiler farm. Therefore, particularly early hatched chicks undergo long periods of fasting, which might last up to the first 72 h of life, in which chicks are able to survive without exogenous nutrition by utilizing energy reserves from their yolk sac (van der Wagt et al., 2020). It has further been shown that even shorter fasting periods of on average 48 h lead to higher mortality rates at 6 wk of age compared to 0 and 24 h of fasting (de Jong et al., 2017). In addition to increased mortality rates, delayed access to feed and water can have negative effects on growth performance (Bigot et al., 2003; Gonzales et al., 2008), development of the immune system (Panda et al., 2015), and susceptibility to diseases in environments with high antigenic pressure (Simon et al., 2015).

Moreover, the absence of early nutrition is only one of several welfare risks associated with conventional hatchery-hatching (**HH**). Exposure to continuous darkness in the hatcher, high noise and dust levels, automated processing on conveyor belts, and transportation may act as stressors that impact chick welfare negatively (Knowles et al., 2004; Archer and Mench, 2017; de Gouw et al., 2017; Hedlund et al., 2019; Giersberg et al., 2020a). Stress at an early age, for instance caused by handling at the hatchery, was found to have long-term consequences on the behavior and welfare of laying hens (Ericsson et al., 2016; Hedlund et al., 2019).

In recent years, alternative hatching systems have been developed to overcome potentially detrimental effects of conventional hatchery practices. One option to prevent post-hatch feed deprivation is to place eggs in special hatchers on day 18 of incubation, in which feed, water, and continuous light are provided (van der Pol et al., 2015). In practice, processing of chicks from these systems also differs from conventional procedures. During processing and transport to the farm, these hatchery-fed (**HF**) chicks remain in the hatching baskets. Another option that also avoids transport of newly hatched chicks is to hatch chicks directly on-farm. In these on-farm hatching systems, eggs are brought from the hatchery to the farm at day 18 of incubation. Currently available on-farm hatching systems differ in the layout and degree of automation (de Jong et al., 2019). In the broiler house, lights are on and feed and water are available upon arrival of the eggs. Automated processing is not required and second grade chicks are usually selected by the farmer. All vaccinations (via drinking water or spray vaccinations) are performed on-farm.

Previous studies have investigated single factors associated with these alternative hatching systems, for instance the absence of transport of newly hatched chicks (Hollemans et al., 2018), continuous darkness in the hatcher (Archer and Mench, 2014), and deprivation of feed and water after hatch (e.g., Bigot et al., 2003; Maiorka et al., 2003; van den Brand et al., 2010). However, limited comparisons between hatching systems have been made with respect to production performance, health, and welfare of broiler chickens early and later in life. de Jong et al. (2019) compared on-farm hatched (**OH**) broiler flocks with conventional hatchery-hatched chickens on commercial farms during the entire production period. They found no effects of the hatching system on performance indicators, such as slaughter weight, feed conversion ratio, and first week and total mortality (de Jong et al., 2019). Under more controlled conditions, on-farm hatching did also not improve body weight and feed conversion ratio until slaughter age but the overall mortality was higher for hatchery-hatched chickens than for OH chickens (de Jong et al., 2020). In both studies, chickens from on-farm hatching systems scored better for the welfare indicator footpad dermatitis (**FPD**, de Jong et al., 2019, 2020). In addition, another welfare aspect, that is the birds' mental welfare, was investigated by Giersberg et al. (2020b), who assessed fearfulness and home-pen behavior of hatchery-hatched and OH chickens. Although the results of the general behavioral observations were ambiguous, hatchery-hatched chickens showed more active and less fearful responses in challenging test situations than OH chickens.

The above-mentioned studies only compared conventional HH with on-farm hatching but did not include a treatment group which had access to feed and water in the hatchery. Although the largest contrast may be expected between conventional HH and on-farm hatching, systems which provide early nutrition and minimal handling in the hatchery might be a good alternative to improve chicken welfare in situations in which on-farm hatching is not feasible. This may for instance be the case in broiler houses in which ambient climatic conditions cannot be adjusted to the requirements of on-farm hatching. Therefore, it should be investigated whether improvements with respect to broiler welfare can also be achieved by these hatchery-feeding systems.

The aim of this study was to assess effects of the hatching environment on the physical and mental welfare of broiler chickens in early and later life by means of various animal-based welfare and behavioral indicators. Chicks from 3 hatching systems were compared: conventional HH, HF with provision of feed and water, and OH. It was hypothesized that a reduction of stressors in the peri- and post-hatching environment, such as the absence of hunger and thirst and less or no handling, would lead to improved welfare both early and later in life. OH chickens were expected to score best, followed by HF chickens, as on-farm hatching does not only include the provision of early nutrition but also the absence of transport of day-old chicks and of other potentially stressful hatchery processing procedures.

## Materials and methods

### Experimental Setup

**Figure 1:**
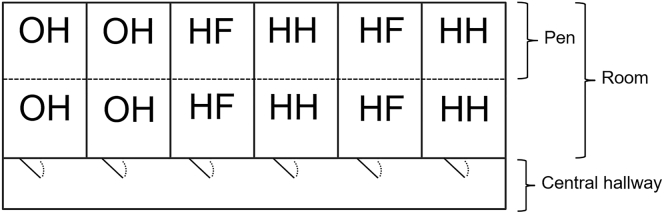
Top view of the broiler house, with rooms for conventionally HH, HF, and OH chickens. Pens were separated by a wire mesh and had separate lines of automated pan feeders and nipple drinkers. Each pen housed 1,155 chickens (batch 1 and 2), and 1,141 chickens (batch 3). Abbreviations: HF, hatchery-fed; HH, hatchery-hatched; OH, on-farm hatched.

The study was carried out from May to October 2019 at the Experimental Poultry Centre in Geel, Belgium. A total of 41,398 Ross 308 chicks was reared in 3 consecutive batches in 6 separate rooms of a broiler house (Figure 1). Each room was accessible from a central hallway and contained 2 adjacent floor pens (each measuring 6.0 × 9.4 m). In the first 2 batches, 1,155 chicks were present per pen at day 0. The third batch started with 1,141 chicks per pen for all treatments. The pens were separated by wire mesh and had separate lines of automated pan feeders and nipple drinkers. Each pen was connected to a central heating system and littered with wood shavings (2–3 cm layer). Within 1 room, chicks from the same hatching system were housed in the 2 pens and per treatment, 2 rooms were used. The location of treatments within rooms or pens did not change between batches as the system for on-farm hatching was installed permanently in 2 rooms and could not be moved. The OH pens were equipped with an X-Treck system (Vencomatic, Eersel, The Netherlands). This system is built of a metal frame which is mounted above a polypropylene belt (33 cm above the floor) that holds the setter trays with the eggs. The chicks fall and dry on the belt after hatching. From the belt, they can reach the floor, and thus feed and water are provided in the pen. After hatching, trays with eggshells and non-hatched eggs were removed and the metal frame was lifted to the ceiling.

All birds were housed according to the European Union law (Council Directive 2007/43/EC, 2007). The experiment was approved by the Institutional Animal Care and Use Committee of the Experimental Poultry Centre on 1 April 2019 (license number EC 2019001). Data on hatching and production performance from this experiment are reported and discussed elsewhere (Souza da Silva, unpublished data).

### Animals and Management Procedures

Within the same batch, chicks from all 3 treatments originated from the same Ross 308 parent stock. The age of the parent flock used for batch 1 was 28 wk, the one used for batch 2 was 29 wk, and the one for batch 3 was 31 wk of age. For the first 18 d, all eggs were incubated at a commercial hatchery (Lagerwey, Lunteren, The Netherlands). At embryonic day (**E**)18, trays were randomly assigned to one of the following hatching systems: conventional HH, HF, or OH. At E18, eggs were candled by a heartbeat system and transferred to hatching baskets (HH and HF) or setter trays (OH).

HH and HF eggs were disinfected twice at E18 and E19 with 37% formaldehyde and further incubated in the hatchery. HH eggs were placed in a conventional hatcher without light, feed, and water. At day 21 of incubation, HH chicks were subjected to standard processing on conveyor belts, including selection of second grade chicks, counting, and placement into transport baskets. Hatching baskets for HF eggs consisted of an egg tray on top of a basket with 2 feed troughs on the sides and holes to reach drinking lines beneath. These baskets were placed in a HatchCare hatcher (HatchTech, Veenendaal, The Netherlands), at E18, which was equipped with light emitting diode lights above open drinking lines on the sidewalls. When chicks hatched, they fell onto the bottom of the basket from which they could reach the feed troughs and the drinking lines. At day 21 of incubation, HF chicks remained in the baskets during the selection of second grade chicks and transportation. During transportation, HF chickens had access to the remaining feed in the feed troughs on the sides of the basket, but not to water. HH and HF chicks were transported separately after 510 and 516 h of incubation, respectively, by conditioned trucks in approximately 2.5 h to the research facility.

Trays with OH eggs were transported by a conditioned truck at E18 to the same research facility and in the same duration. After arrival at the research facility, the trays were set in the X-Treck system (see above). At day 21 of incubation, non-hatched eggs were removed from the OH pens and second grade chicks were selected by the animal caretakers and culled humanely. Continuous light was provided in the OH pens starting at E18. The light regime started at the day of arrival of HH and HF chicks with 1D:23 L (day 0) and was gradually reduced to 3L:1D:12L:1D:3L:4D (day 7 and onward). During the final days before slaughter (day 38–40), the initial light regime of 1D:23 L was maintained. The ambient temperature of the OH treatment was adjusted based on eggshell temperature (E18-day 0). From day 2, the ambient temperature was gradually decreased from approximately 34°C to 19°C at day 40. A commercially available 4-phase diet was provided ad libitum, which was supplied on chick paper during the first days, starting at E18 in the OH pens. Thinning was performed at day 33 by removing 280 broilers from each pen for slaughter. At 40 d of age, the remaining chickens were sent to a commercial slaughter plant. For detailed management procedures, please also see the work of Souza da Silva, unpublished data.

### Welfare Assessment

In order to evaluate the welfare of the chickens from the different hatching systems, several indicators based on the birds' environment, health, and behavior were assessed at different time points during the rearing period. In order to minimize the effects of disturbance on the birds' responses, behavioral tests were carried out first in case they were performed on the same study day as environmental and animal-based welfare measurements. The pens were visited in a random order on each assessment day. Two observers measured the environmental indicators. Data on animal-based welfare indicators were collected by 1 observer, except for walking ability, which was scored by 2 observers. All behavioral tests were conducted by 3 observers.

#### Environmental Indicators

Environment-based welfare indicators were assessed at 21 and 35 d of age. Litter quality was scored visually according to the Welfare Quality Assessment Protocol for Poultry (2009) on a 5-point scale ranging from 0 (completely dry and flaky) to 4 (sticks to boots once the cap or compacted crust is broken) in 3 locations per pen (near the wall, near the feeders and drinkers, and in the central litter area). The NH_3_ level was measured at birds' height in the middle of each pen with Kitagawa AP-1 Precision Gas Detector (Komyo, Kawasaki-City, Japan).

#### Animal-based Welfare Indicators

At 21 and 35 d of age, a random sample of 30 chickens per pen (n = 120 birds per hatching system) was enclosed in a catching pen. Each bird was assessed individually for breast blisters (score 0 = no discoloration, lesion, or blister to 2 = blister or lesion, skin open, often covered with crust), plumage cleanliness (0 = feathers and skin are completely clean to 3 = dirt is caked on the feathers or skin of the belly and also the rest of the plumage is visibly dirty), skin lesions (0 = no lesions or single punctiform damage to 2 = at least 1 lesion ≥2 cm diameter at the largest extent), FPD (0 = no lesions to 4 = ulcers or scabs, signs of hemorrhages, or deep dermatitis), and hock burn (0 = no lesions to 4 = brown or black discoloration of the hock, more than 1 spot possible, total >0.5 cm^2^, probably with crust; also infected hock). All these scoring schemes followed the Welfare Quality Assessment Protocol for Poultry (2009). As breast discoloration occurred only in 1 chicken during the entire study, this score was not included in further analysis. In addition, the walking ability of another 30 birds per pen was evaluated at day 35 according to the gait score scheme (0 = normal, dexterous, and agile to 5 = incapable of walking) of the Welfare Quality Assessment Protocol for Poultry (2009).

#### Animal-based Behavioral Indicators

A novel environment (**NE**) test was carried out according to a protocol by de Haas et al. (2014) at 1 and 21 d of age. Five chickens per pen (n = 20 birds per treatment) were individually caught and transported in a black bucket to the test location in the central hallway. After the bird was placed in a gray, non-transparent plastic box (62 × 38 × 36 cm), which served as the NE, its response was recorded for 2 min (latency to vocalize, number of vocalizations, and number of flight attempts). The variable ‘number of flight attempts’ was not subjected to statistical analyses because only 16 out of 360 chickens showed an attempt to escape.

At 4 and 35 d of age, 5 birds per pen were subjected to a tonic immobility (**TI**) test as described by Hollemans et al. (2018). Each chicken was taken from its home pen and transported in a black bucket to the test location. There, it was placed in a metal cradle and restrained on its back for 10 s by the experimenter, who used 1 hand to hold its chest and 1 hand to cover its neck and head. Eye contact with the bird was avoided. If the chicken stood up within 10 s after restraining, TI was induced again, up to a maximum of 5 times. After 5 attempts, the test was stopped, and a missing value was recorded. If the chicken remained on its back for at least 10 s after restraining, it was judged immobile and the latency (s) until standing up was recorded. The test was terminated at a maximum latency of 300 s. As the latency to stand up of older broilers may not only be influenced by fear but also by the incapability to turn around due to their high body weight, the birds' first attempt to erect themselves (i.e., latency to first wing or leg movement) was additionally recorded at day 35 and the test was terminated after 180 s.

The response to a novel object (**NO**) was tested according to a procedure of de Haas et al. (2014) and the Welfare Quality Assessment Protocol for Poultry (2009). At 4 d of age, a wooden block (8 × 5 × 2.5 cm) wrapped with colored tape (green, white, red, black, and blue) served as the NO. The test lasted for 120 s during which the number of chickens within a 25 cm radius of the NO was recorded every 10 s. In addition, the latency for the first 3 birds to approach the NO (<25 cm) was measured. At day 35, the test was repeated using a round plastic stick (50 cm in length, 2.5 cm in diameter) wrapped with colored tape as the NO.

An avoidance distance (**AD**) test according to the Welfare Quality Assessment Protocol for Poultry (2009) was carried out at 4 and 35 d of age. The observer approached a group of at least 3 chickens in the pen, squatted for 10 s, and counted the number of birds within a semicircle of <1 m in front of her. The test was repeated at 6 locations per pen.

### Statistical Analyses

All statistical analyses were performed using the SPSS Statistics software (version 25, IBM, Armonk, NY). All data were assessed for normal distribution by creating histograms including the Gaussian distribution curve. Homogeneity of variance was tested according to the Levene procedure. The experimental unit was the pen. Generalized linear mixed models consisted of environmental indicators, animal-based welfare indicators, and responses in the behavioral tests as target variables and the fixed effects of hatching system, age, the interaction between the hatching system and age, and testing order. Room and batch were added as block effects. For visual litter scores and animal-based welfare indicators, models were fitted with a multinomial probability distribution and a generalized logit link function. Generalized linear mixed models with a normal distribution and a log link function were used for NH_3_ levels and all behavioral responses, except for vocalizations in the TI test, for which a binomial distribution with a logit link function was chosen. Observer was added to the model as a fixed effect for the behavioral tests. Fixed effects with *P* > 0.1 (i.e., testing order and observer) were excluded in the final models by means of a backward regression procedure. Post hoc pairwise comparisons were adjusted by Bonferroni correction. *P*-values < 0.05 were considered to be statistically significant.

## Results

### Effects of Hatching System and Age on Environmental Indicators

At day 21, a visual litter score of 3 (‘sticks to boots and sticks readily in a ball if compacted’) was given in 43% of the cases, followed by a score of 2 (39%), 4 (11%), 1 (6%), and 0 (1%). At day 35, the most frequently assigned score was 3 (58%), followed by 4 (19%), 2 (16%), and 1 (7%). There was neither a significant age × hatching system interaction for visual litter score, nor an effect of the hatching system. Higher, and thus worse, litter scores were recorded at day 35 compared to day 21 (F_4,44_ = 3.52, *P* < 0.05). Average NH_3_ levels were 1.64 ppm at day 21 and 0.89 ppm at day 35. Again, there was only an effect of age, with higher NH_3_ levels measured at day 21 compared to day 35 (F_1,66_ = 37.39, *P* < 0.001).

### Effects of Hatching System and Age on Animal-Based Welfare Indicators

**Figure 2:**
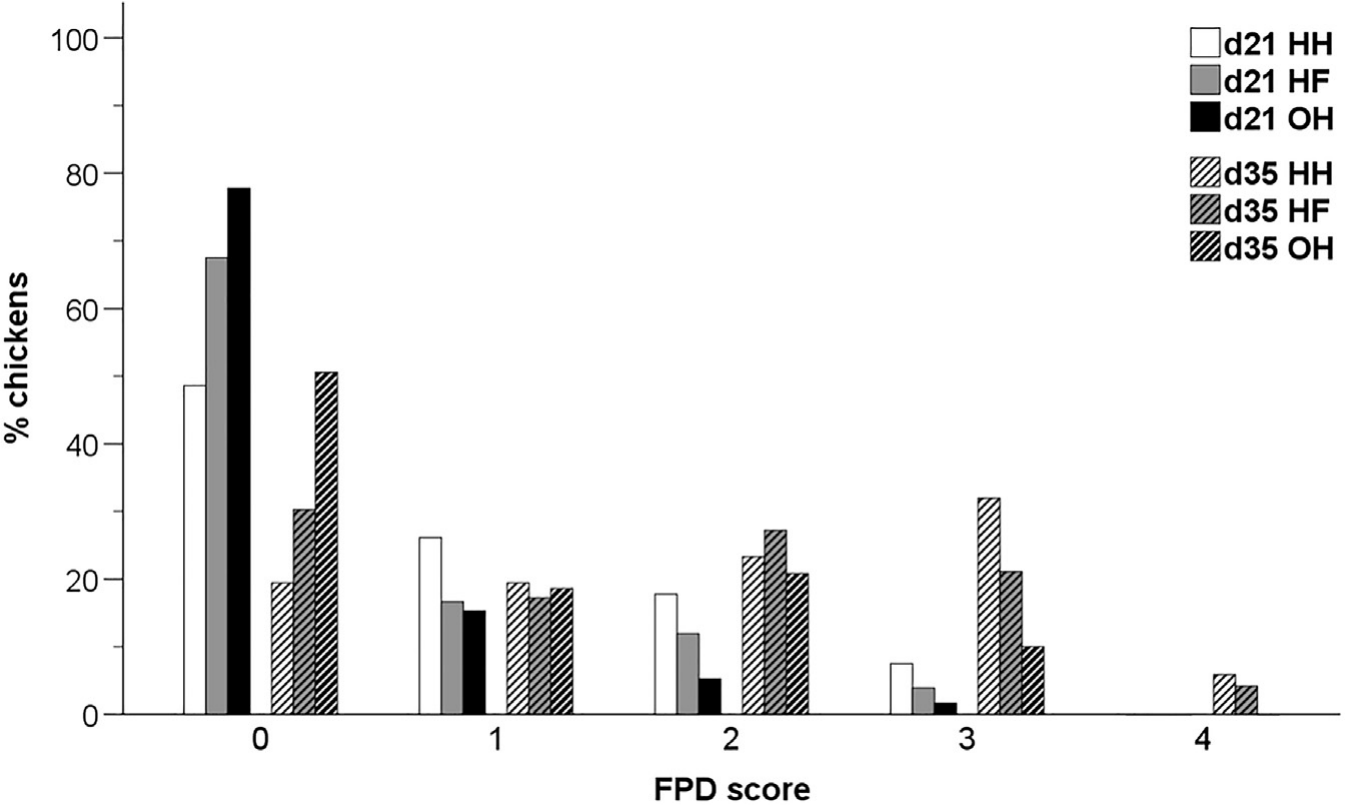
Distribution of FPD scores for conventionally HH, HF, and OH broiler chickens at day 21 and 35 of age. A higher score indicates more severe lesions. FPD scores were significantly affected by the hatching system (*P* < 0.05) and age (*P* < 0.001). Abbreviations: FPD, footpad dermatitis; HF, hatchery-fed; HH, hatchery-hatched; OH, on-farm hatched.

There were no significant age × hatching system interactions for the indicators plumage cleanliness, skin lesions, FPD, and hock burn. An effect of hatching system was only found for FPD (F_4,44_ = 3.05, *P* < 0.05), with HH chickens having more severe footpad lesions compared to HF (*P* < 0.05) and OH chickens (*P* < 0.01), and HF chickens scoring worse than OH chickens (*P* < 0.05) (Figure 2). Significant effects of age were found for all of the assessed animal-based welfare indicators. Higher scores, which indicate worse quality, were observed for plumage cleanliness (F_3,51_ = 66.44, *P* < 0.001), skin lesions (F_2,58_ = 160.40, *P* < 0.001), FPD (F_4,44_ = 62.98, *P* < 0.001), and hock burn (F_4.44_ = 75.47, *P* < 0.001) at day 35 compared to day 21 (supplementary material, Supplementary Figure 1). Gait score, which was assessed at day 35, did not differ among the investigated hatching systems (F_6,27_ = 0.55, *P* = 0.77) (supplementary material, Supplementary Figure 1).

### Effects of Hatching System and Age on Animal Behavior

**Figure 3:**
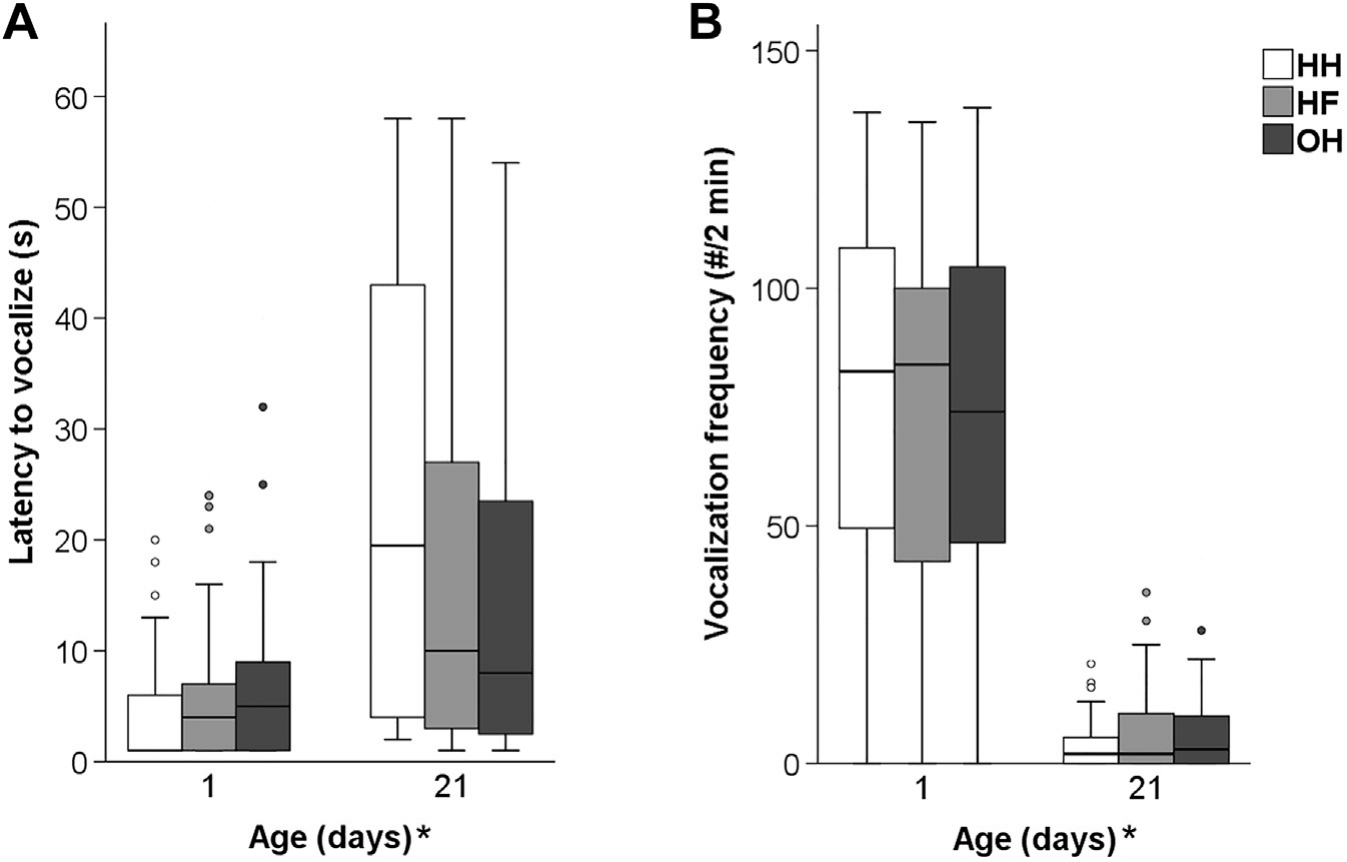
Responses in the NE test at 1 and 21 d of age (n = 60 chickens per hatching system): (A) mean latency to vocalize (±SEM), (B) mean vocalization frequency (±SEM) of conventionally HH, HF, and OH broiler chickens. ∗After ‘Age (d)’ denotes an age effect (*P* < 0.05). Abbreviations: HF, hatchery-fed; HH, hatchery-hatched; NE, novel environment; OH, on-farm hatched.

In the NE test, no significant age × hatching system interactions were found for the latency to vocalize and number of vocalizations. In addition, both variables did not differ between hatching systems. Age affected the latency to vocalize (F_1,288_ = 40.97, *P* < 0.001) and number of vocalizations (F_1,354_ = 66.46, *P* < 0.001). The chickens vocalized sooner and more frequently at day 1 compared to day 21 (Figure 3).

**Figure 4:**
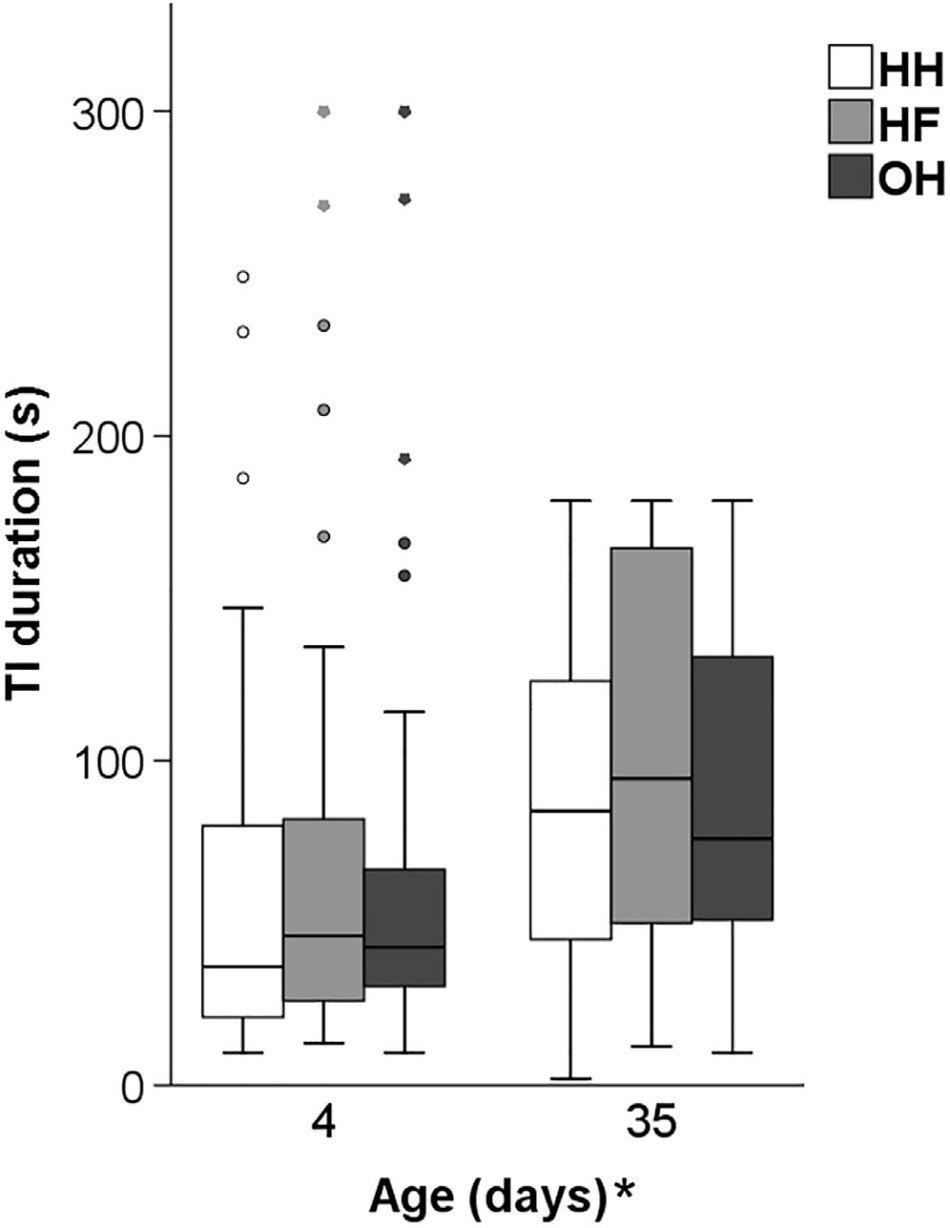
Mean TI durations (±SEM) in the TI test at 4 and 35 d of age (n = 60 chickens per hatching system) of conventionally HH, HF, and OH broiler chickens. ∗After ‘Age (d)’ denotes an age effect (*P* < 0.05). Abbreviations: HF, hatchery-fed; HH, hatchery-hatched; OH, on-farm hatched; TI, tonic immobility.

**Figure 5:**
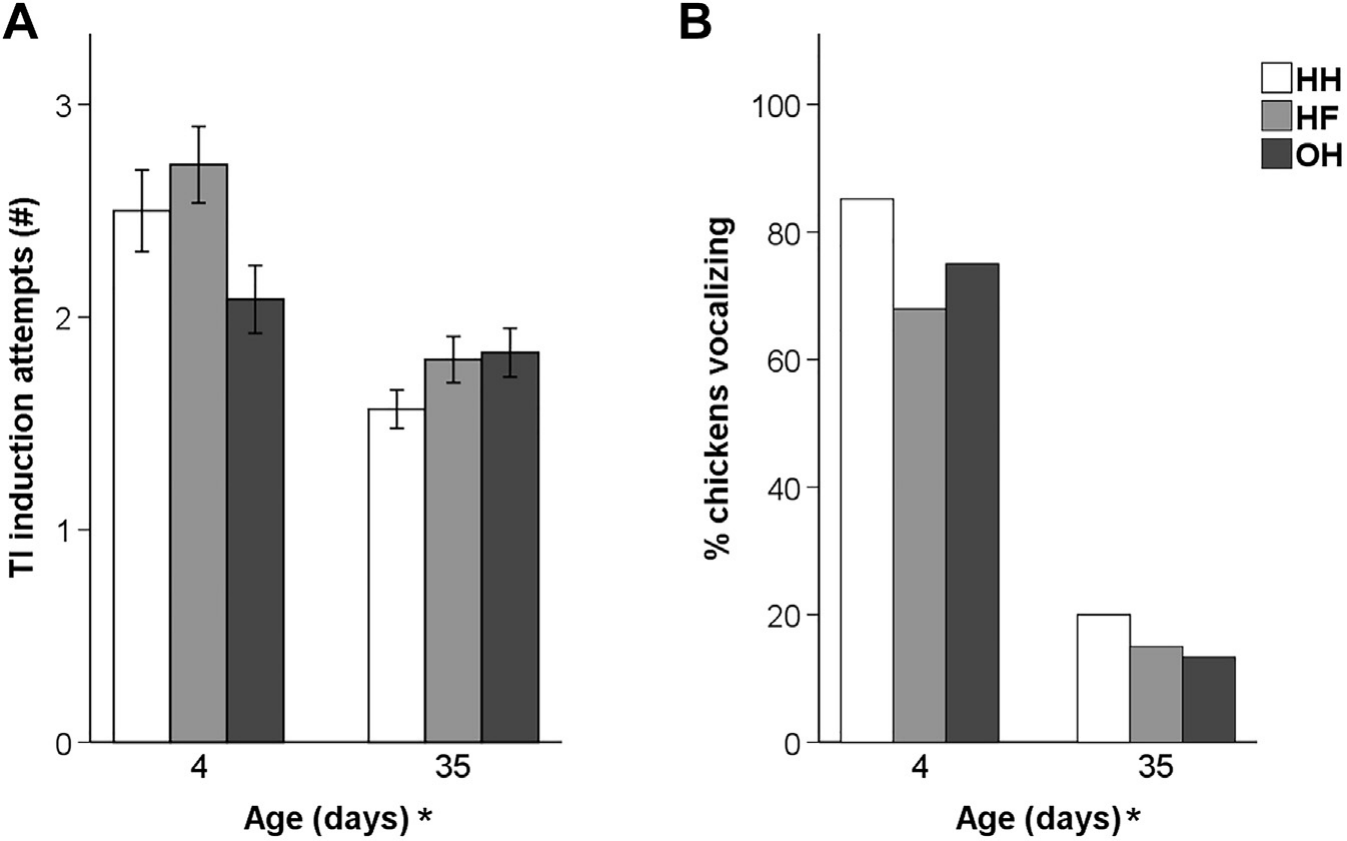
Responses in the TI test at 4 and 35 d of age (n = 60 chickens per hatching system): (A) mean number (±SEM) of TI induction attempts, (B) percentage of chickens vocalizing during TI of conventionally HH, HF, and OH broiler chickens. ∗After ‘Age (d)’ denotes an age effect (*P* < 0.05). Abbreviations: HF, hatchery-fed; HH, hatchery-hatched; OH, on-farm hatched; TI, tonic immobility.

Mean TI durations in all groups were <80 s at day 4 and <100 s at day 35 (Figure 4). Neither age × hatching system interactions, nor effects of the hatching system were found. TI durations were significantly longer at day 35 than at day 4 (F_1,337_ = 9.52, *P* < 0.01). Similarly, the number of TI induction attempts and the proportion of chickens which vocalized during the test differed between 4 and 35 d of age (Figure 5). At day 4, more attempts were needed to induce TI (F_1,354_ = 39.93, *P* < 0.001) and more chickens vocalized (F_1,337_ = 103.93, *P* < 0.001) compared to day 35. Again, there were no significant age × hatching system interactions and no effects of the hatching system.

**Figure 6:**
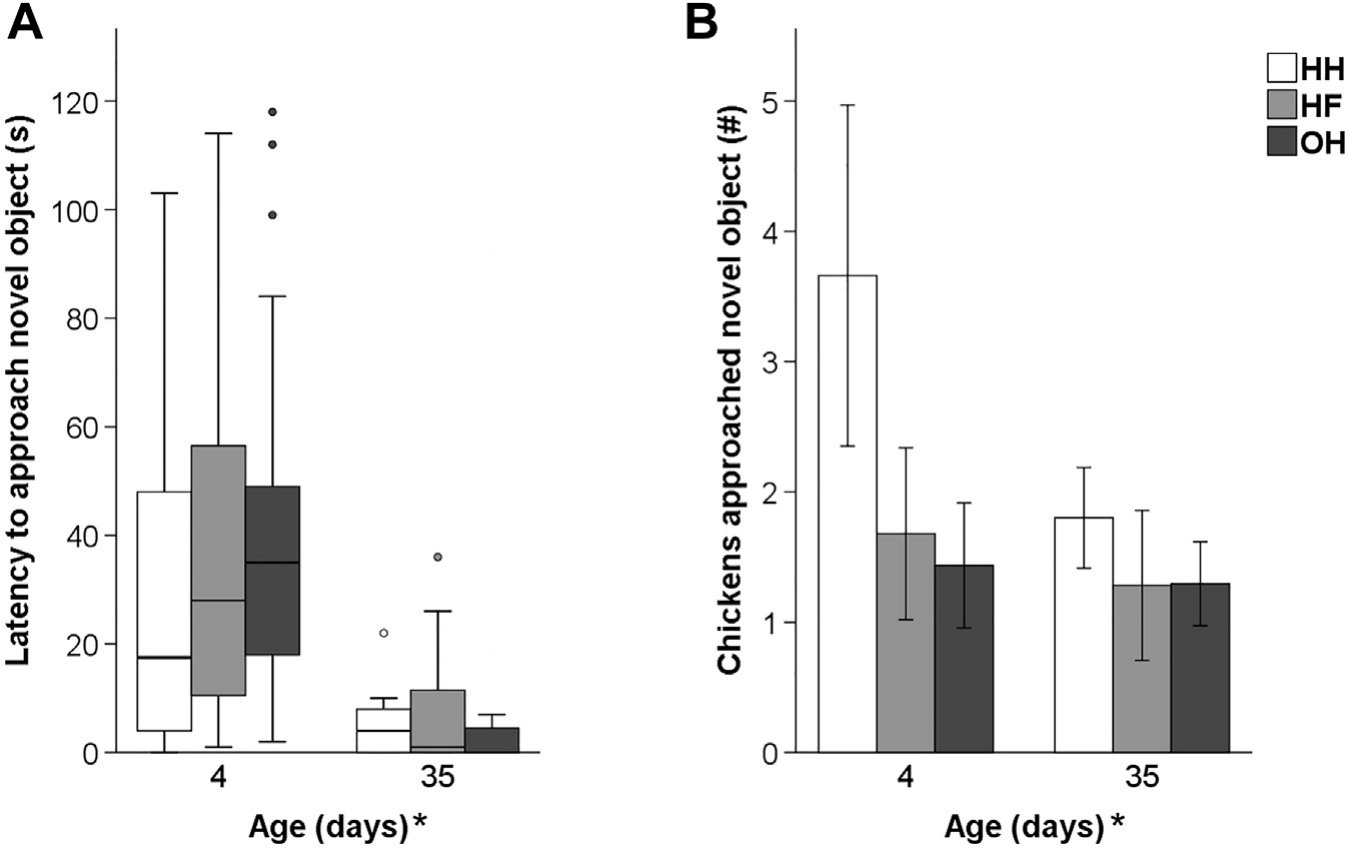
Responses in the NO test at 4 and 35 d of age (n = 12 pens per hatching system): (A) mean latency (±SEM) of the first 3 chickens to approach, (B) mean number (±SEM) of chickens approaching (<25 cm) the NO of conventionally HH, HF, and OH broiler chickens. ∗After ‘Age (d)’ denotes an age effect (*P* < 0.05). Abbreviations: HF, hatchery-fed; HH, hatchery-hatched; NO, novel object; OH, on-farm hatched.

In the NO test, there were no age × hatching system interactions, and no significant differences between HH, HF, and OH chickens. Age effects were found for latency of the first 3 chickens to approach (F_1,66_ = 20.91, *P* < 0.001) and for the average number of birds within a 25-cm radius of the NO (F_1,66_ = 5.95, *P* < 0.05). A higher number of chickens approached the NO at day 4, but they approached it sooner at day 35 (Figure 6).

**Figure 7:**
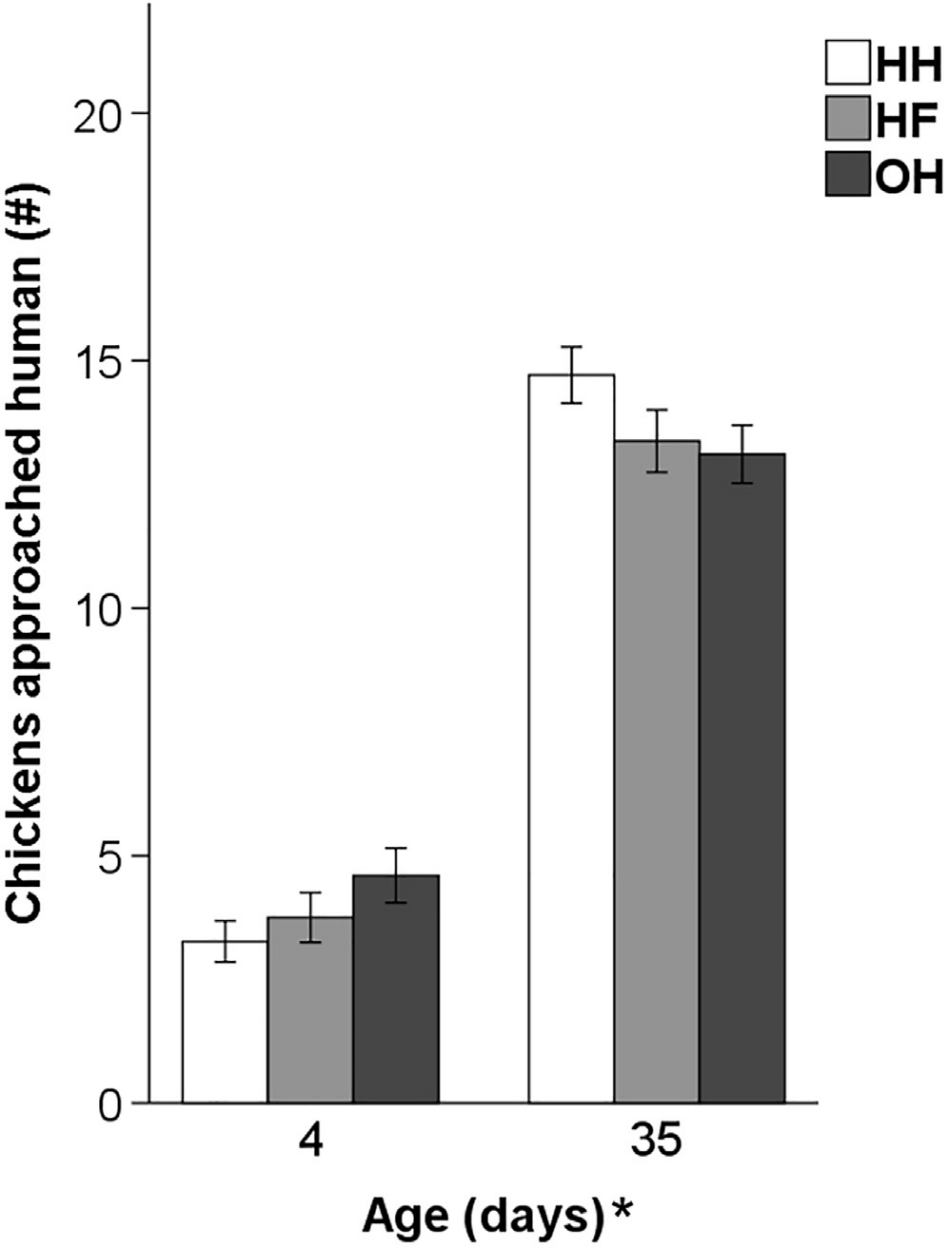
Mean number (±SEM) of chickens approaching a human (semicircle, <1 m) in the AD test at 4 and 35 d of age (n = 12 pens per hatching system) of conventionally HH, HF, and OH broiler chickens. ∗After ‘Age (d)’ denotes an age effect (*P* < 0.05). Abbreviations: AD, avoidance distance; HF, hatchery-fed; HH, hatchery-hatched; OH, on-farm hatched.

The interaction of age × system and hatching system had no effect on the response in the AD test. Age affected the average number of chickens found within a semicircle of <1 m of the observer (F_1,66_ = 250.28, *P* < 0.001). More chickens approached the observer at day 35 compared to day 4 (Figure 7).

## Discussion

The aim of the present study was to assess the effects of 3 hatching systems on the welfare of broiler chickens early as well as later in life. In general, all animal-based welfare and behavioral indicators were affected by age, whereas the hatching system had an effect on FPD scores only. Significant interactions between age and hatching system were not found either.

It is important to note that the current study was designed as a system comparison. HH, HF, and OH hatching systems are characterized by several distinct features, which may affect the chickens in various ways. It is therefore not possible to draw conclusions about single influencing factors, such as hatchery processing, transportation of eggs or day-old chicks, or the presence or absence of early feeding. The 3 different hatching systems and the associated management procedures applied here are available for commercial use. As the chickens were observed in systems and social contexts similar to those found on commercial farms, the present results are applicable for practical settings as well.

In general, litter quality was slightly poorer compared to earlier findings on commercial farms (de Jong et al., 2019). NH_3_ levels in all pens were well below the legal limit of 20 ppm (Council Directive 2007/43/EC, 2007). Both environmental indicators did not differ among hatching systems. Using the same scoring method for litter quality, de Jong et al. (2019) found a tendency for better scores in OH compared to HH flocks when collecting data of commercial farms at day 21 and 39. However, under more controlled conditions at an experimental farm, weekly recorded visual litter quality did not differ between pens with HH and pens with OH chickens (de Jong et al., 2020).

The significantly higher prevalence and severity of FPD in HH compared to HF, and in HF compared to OH chickens might therefore not be explained by differences in litter quality. However, litter moisture content, which was not measured in the present study, has been shown to vary among pens with the same visual litter score (de Jong et al., 2020). Increased litter moisture is associated with more severe FPD (Shepherd and Fairchild, 2010). In line with this, de Jong et al. (2020) observed both a higher litter moisture content and a higher prevalence of footpad lesions in pens with HH chickens than in those with OH chickens. The underlying causes for these findings remained unclear, as no long-term differences of intestinal development or diseases, which could alter feces quality, and thus litter moisture level, were found between HH and OH chickens (de Jong et al., 2020). Although previous studies reported inconsistent results, body weight may also influence the occurrence of footpad lesions, with heavier chickens being more likely to develop FPD (Shepherd and Fairchild, 2010). However, in the present study, OH chickens were heavier than HH chickens throughout the production period, with HF chickens having intermediate weights (Souza da Silva, unpublished data), which does not explain the differences in FPD scores. Further animal-based welfare indicators, that is plumage cleanliness, skin lesions, and hock burn scored significantly worse with increasing age but were not affected by the hatching system. Similar results were obtained when comparing OH and HH, under both commercial and experimental conditions (de Jong et al., 2019, 2020). Gait score, which was only assessed at day 35, was not affected by the hatching system, which is also in line with previous studies (de Jong et al., 2019, 2020).

Apart from environment-based welfare indicators and body condition scoring, the chickens' behavior was studied to evaluate mental welfare (Dawkins, 1999). As results of previous general behavioral observations in the home pens of HH and OH chickens were ambiguous (Giersberg et al., 2020b), chickens from the 3 hatching systems were only observed in more challenging test situations in the current study and a set of validated fear tests (Forkman et al., 2007) was carried out. Measuring fear-related responses does not require prior training of animals, which would not be feasible in newly hatched chicks, and which might overshadow treatment effects by acting as positive enrichment itself. However, contrary to our hypothesis, no effect of hatching system was observed that would indicate reduced fearfulness in OH or HF compared to HH chickens.

In the NE test, flight attempts occurred rarely, and latency to vocalize and vocalization frequency were only affected by age, contrary to the results of previous studies. In a study by Giersberg et al. (2020b), HH chicks showed more flight attempts than OH chicks at day 1 and 8 and therefore seemed to act less fearful (Forkman et al., 2007). In addition, HH chicks vocalized more at day 1 and less at day 8 compared to OH chicks (Giersberg et al., 2020b). In contrast, Hedlund et al. (2019) found more fearful and less active responses in a similar test situation in day-old layer chicks, which were—similar to HH chicks—processed in a commercial hatchery, compared to chicks which hatched at a research facility and were not handled. The discrepancy between those studies remains unclear, but might be related to differences between layer and broiler chicks, as layer chicks were often found to show more vigorous responses in fear tests than broiler chicks (Keer-Keer et al., 1996). Furthermore, the differences may also be explained by differences in parent stock age, which was not specified by Hedlund et al. (2019).

The increase in the latency to vocalize and the decrease of vocalization frequency with age in the present study are supported by previous results. The responses of chickens in the NE test can be regarded as a compromise between avoiding predation (not vocalizing) and regaining social contact (vocalizing immediately and frequently) (Suarez and Gallup, 1983; Marx et al., 2001). In chickens at a young age, seeking social reinstatement seems to predominate, but it becomes less important with aging (Suarez and Gallup, 1983).

Longer durations of TI have been related to higher levels of fearfulness in chickens (Forkman et al., 2007). In contrast to our results, Hollemans et al. (2018) found longer TI durations in transported, early-fed chickens compared to transported, delayed-fed chickens when tested at 3 d of age but reverse effects at day 30. This was explained by a possibly delayed cognitive development in the feed-deprived chickens, and thus a lack of ability to express fear-related responses at an early age (Hollemans et al., 2018). However, all chicks in that study hatched in small experimental pens before simulated transport and were not subjected to commercial processing procedures, which may explain the different results compared to the present study. The age effects observed in chickens from all 3 hatching systems in the TI test in the current study correspond to earlier findings. It has been shown that although TI induction at a very young age is possible, TI durations increase sharply at 5 d of age (Heiblum et al., 1998), which would be after our first tests at day 4. It is unlikely that longer TI durations found at day 35 in the current study were influenced by the incapability of heavier broilers to erect themselves, as first wing or leg movements were recorded and regarded as termination of TI at this age as well. The higher number of chickens vocalizing at day 4 compared to day 35 can again be explained by the high motivation of seeking social reinstatement by emitting ‘calling sounds’ at a young age (Heiblum et al., 1998; Fontana et al., 2016).

Shorter latencies to approach a NO indicate reduced fearfulness at day 35 compared to day 4 (Forkman et al., 2007). In this respect, one would expect that also more chickens would approach NO at an older age, which was not the case in the present study. However, a major challenge in interpreting the NO test is that both fearful and indifferent groups of animals may not approach the NO (Forkman et al., 2007). Therefore, the decrease in the latency to approach and the numbers of chickens near the NO at day 35 might be explained by a decrease in fearfulness in those chickens that approached it, accompanied by a general lack of interest of the group. As walking in general decreases in fast growing broilers from 3 wk of age onward (Bokkers and Koene, 2003), the NO might not have been a stimulus strong enough to elicit such an activity in the chickens. In the AD test, all chickens within a semicircle of <1 m in front of the observer were recorded, that is both chickens that approached her and those that did not withdraw. Therefore, chickens at day 35 showed less fear of humans than at day 4, either by actively approaching or by not moving away.

Results of the behavioral tests showed that patterns of habituation or familiarization leading to reduced fearfulness with increasing age are present in all groups of chickens, independent of the hatching system and in line with previous studies (Giersberg et al., 2020b). As it was not possible to identify individual birds in the present study, it is not known whether the same or different individuals were tested at a young and an older age, and how this varied among the 3 hatching systems. However, a learning effect of individual chickens seems unlikely, as there were 20 to 31 d between the 2 testing times, during which no training or testing was performed. It remains difficult to interpret why HH chickens expressed less fearful and more active responses than OH chickens in similar test situations in a previous study (Giersberg et al., 2020b). As mentioned earlier, both studies represent a system comparison with several factors which were kept constant over batches within 1 study but which differed between the studies. The HH chicks observed by Giersberg et al. (2020b), for instance, were hatched and processed in a different commercial hatchery than those in the present study. Moreover, the chicks in Giersberg et al. (2020b) originated from older parent stocks (35–41 wk) and HH chicks were subjected to shorter transport durations (approximately 45 min). It has been shown that there is variation in post-hatch processing procedures and in physical severity of handling across commercial hatcheries (Knowles et al., 2004). It is further not known at which exact times the chickens had first contact with humans. For hatchery-hatched chickens, this depends on the hatch window: early hatched chickens will have contact with humans during processing relatively later in their lives than late hatched chickens. In the on-farm hatching system, first contact with humans depends on the exact hatching time of the individual relative to the routine inspection rounds of the caretaker. Therefore, differences in incubation conditions and handling procedures in the different hatcheries and on the different farms might have led to differences in the behavior between HH and OH chickens.

It may also be possible that the hatching system had more subtle effects on the mental welfare of the chickens, which could not be detected in the behavioral tests. Physiological stress indicators, such as basal levels of corticosterone and corticosterone reactivity (Ericsson et al., 2016; Hedlund et al., 2019), remain to be investigated in chickens hatched in the 3 different hatching systems.

To our knowledge, this is the first study comparing the effects of 3 different, commercially available hatching systems on the welfare of broiler chickens during the entire production period. Conventional hatchery-hatched chickens scored significantly worse for the key indicator FPD, whereas, in general, hatching system seemed to have minor effects on the physical and mental welfare of broiler chickens early and later in life. However, to which extent the effects of the hatching system interact with the specific management procedures of the respective commercial facility and how parent stock age plays a role merits further study.

## Supplementary data

Supplementary Figure 1 Distribution of gait (A), plumage cleanliness (B), skin lesions (C), and hock burn (D) scores for conventionally hatchery-hatched (HH), hatchery-fed (HF), and on-farm hatched (OH) broiler chickens at 1 and day 35 of age. A higher score indicates a worse quality.
