# Supplementary figures and images for: Effects of hatching system on the welfare of broiler chickens in early and later life
Source: Poult Sci. 2020 Dec 23;100(3):100946. doi: 10.1016/j.psj.2020.12.043 (PMC7936212; doi:10.1016/j.psj.2020.12.043)

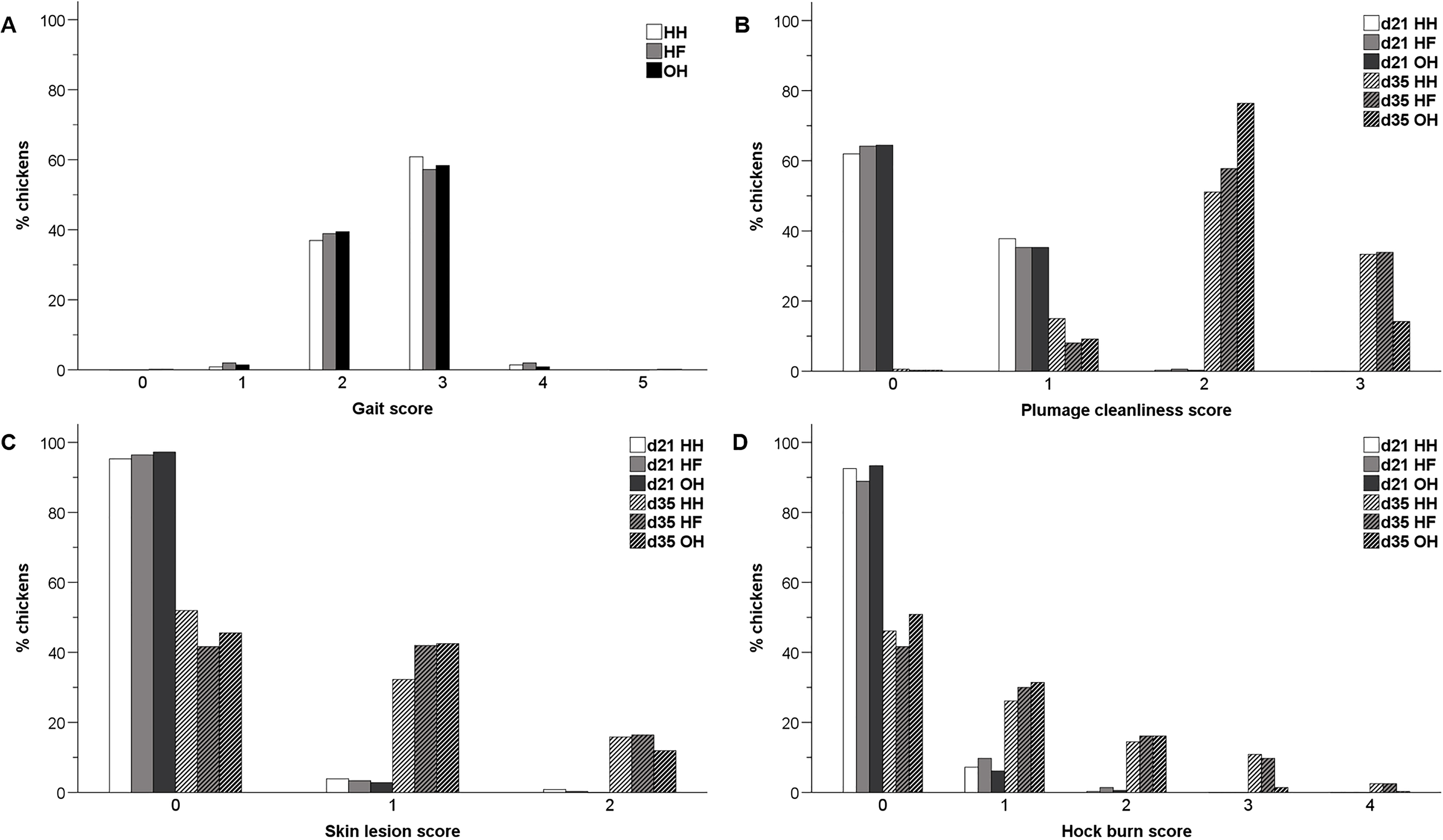

Supplement: Supplementary Figure 1 — Distribution of gait (A), plumage cleanliness (B), skin lesions (C), and hock burn (D) scores for conventionally hatchery-hatched (HH), hatchery-fed (HF), and on-farm hatched (OH) broiler chickens at 1 and day 35 of age. A higher score indicates a worse quality. [file figs1.jpg]
